# Emotional Exhaustion Among US Health Care Workers Before and During the COVID-19 Pandemic, 2019-2021

**DOI:** 10.1001/jamanetworkopen.2022.32748

**Published:** 2022-09-21

**Authors:** J. Bryan Sexton, Kathryn C. Adair, Joshua Proulx, Jochen Profit, Xin Cui, Jon Bae, Allan Frankel

**Affiliations:** 1 Department of Psychiatry, Duke University School of Medicine, Durham, North Carolina; 2 Duke Center for Healthcare Safety and Quality, Duke University Health System, Durham, North Carolina; 3 Duke University Health System, Durham, North Carolina; 4 Safe & Reliable Healthcare, LLC, Evergreen, Colorado; 5 Division of Neonatal and Developmental Medicine, Department of Pediatrics, Stanford University School of Medicine and Lucile Packard Children’s Hospital, Palo Alto, California; 6 California Perinatal Quality Care Collaborative, Palo Alto, California; 7 Department of Medicine, Duke University School of Medicine, Durham, North Carolina

## Abstract

**Importance:**

Extraordinary strain from COVID-19 has negatively impacted health care worker (HCW) well-being.

**Objective:**

To determine whether HCW emotional exhaustion has increased during the pandemic, for which roles, and at what point.

**Design, Setting, and Participants:**

This survey study was conducted in 3 waves, with an electronic survey administered in September 2019, September 2020, and September 2021 through January 2022. Participants included hospital-based HCWs in clinical and nonclinical (eg, administrative support) roles at 76 community hospitals within 2 large health care systems in the US.

**Exposures:**

Safety, Communication, Organizational Reliability, Physician, and Employee Burnout and Engagement (SCORE) survey domains of emotional exhaustion and emotional exhaustion climate.

**Main Outcomes and Measures:**

The percentage of respondents reporting emotional exhaustion (%EE) in themselves and a climate of emotional exhaustion (%EEclim) in their colleagues. Survey items were answered on a 5-point scale from 1 (strongly disagree) to 5 (strongly agree); neutral or higher scores were counted as “percent concerning” for exhaustion.

**Results:**

Electronic surveys were returned by 37 187 (of 49 936) HCWs in 2019, 38 460 (of 45 268) in 2020, and 31 475 (of 41 224) in 2021 to 2022 for overall response rates of 74.5%, 85.0%, and 76.4%, respectively. The overall sample comprised 107 122 completed surveys. Nursing was the most frequently reported role (n = 43 918 [40.9%]). A total of 17 786 respondents (16.9%) reported less than 1 year at their facility, 59 226 (56.2%) reported 1 to 10 years, and 28 337 (26.9%) reported 11 years or more. From September 2019 to September 2021 through January 2022, overall %EE increased from 31.8% (95% CI, 30.0%-33.7%) to 40.4% (95% CI, 38.1%-42.8%), with a proportional increase in %EE of 26.9% (95% CI, 22.2%-31.8%). Physicians had a decrease in %EE from 31.8% (95% CI, 29.3%-34.5%) in 2019 to 28.3% (95% CI, 25.9%-31.0%) in 2020 but an increase during the second year of the pandemic to 37.8% (95% CI, 34.7%-41.3%). Nurses had an increase in %EE during the pandemic’s first year, from 40.6% (95% CI, 38.4%-42.9%) in 2019 to 46.5% (95% CI, 44.0%-49.1%) in 2020 and increasing again during the second year of the pandemic to 49.2% (95% CI, 46.5%-51.9%). All other roles showed a similar pattern to nurses but at lower levels. Intraclass correlation coefficients revealed clustering of exhaustion within work settings across the 3 years, with coefficients of 0.15 to 0.17 for emotional exhaustion and 0.22 to 0.24 for emotional exhaustion climate, higher than the .10 coefficient typical of organizational climate (a medium effect for shared variance), suggestive of a social contagion effect of HCW exhaustion.

**Conclusions and Relevance:**

This large-scale survey study of HCWs spanning 3 years offers substantial evidence that emotional exhaustion trajectories varied by role but have increased overall and among most HCW roles since the onset of the pandemic. These results suggest that current HCW well-being resources and programs may be inadequate and even more difficult to use owing to lower workforce capacity and motivation to initiate and complete well-being interventions.

## Key Points

**Question:**

Is the COVID-19 pandemic associated with an increase in health care worker emotional exhaustion?

**Findings:**

In this 3-year survey study with an overall sample of 107 122 responses from US health care workers before (2019) and twice during (2020 and 2021-2022) the COVID-19 pandemic, increases were reported in assessments of emotional exhaustion in oneself and in one’s colleagues overall and for every role; nurses reported increases each year, but physicians reported decreases in 2020 followed by sharp increases in 2021. Exhaustion score clustering in work settings was suggestive of a social contagion effect of exhaustion.

**Meaning:**

These findings indicate that emotional exhaustion among health care workers, which was problematic before the pandemic, has become worse; increases in emotional exhaustion may jeopardize care quality and necessitate additional support for the workforce.

## Teaser

This survey study examines changes in health care workers’ reported emotional exhaustion before and during the COVID-19 pandemic.

## Introduction

The challenges posed by COVID-19 have been an excessive test to human well-being around the world. Few groups experienced this stress more acutely than the health care workers (HCWs) who persistently placed themselves in harm’s way to serve patients. Early during the pandemic, HCWs were recognized as heroes facing the uncertainty of a new health crisis. Unique demands for remote learning, working, and child care required adaptation, as did uncertainty around available resources, evolving visitation policies, accessibility of vaccines, consequences of vaccine mandates, and the overt politicization of public health during historically tumultuous times.

The Delta and Omicron surges then delivered high proportions of unvaccinated patients and their family members to HCWs who had endured emotional and physical exhaustion for 18 months. Simultaneously, HCWs experienced acute staffing shortages and an intensification of incivility directed at them for following protocols intended to keep everyone safe. Health care workers being accosted and accused of conspiracies was not uncommon. Moral injuries of this kind have been described as the negative impact of exposure to transgressions of deeply held moral beliefs and expectations.^1^ Moral injury in HCWs is consistently associated with lower quality of life and higher levels of emotional exhaustion.^2,3^ Perhaps it should not be a surprise that 40% of nurses and 23.8% of physicians plan to exit their practice in the next 2 years.^4^ The pandemic has taken a toll on HCW mental health, and the story is still unfolding.

In the first year of the pandemic, prior to the Delta and Omicron surges, a systematic review and meta-analysis^5^ of HCW mental health identified a high prevalence (22%) of moderate depression, anxiety, and posttraumatic stress disorder. A comparison of post-9/11 combat veterans to HCWs during the pandemic demonstrated roughly equivalent rates of moral injury in both groups.^6^ For combat veterans, risk increased with exposure to post-battle traumatic experiences, but for HCWs, the increase came with working in higher-risk COVID-19 units and exposure to COVID-19. The disproportionate psychological, emotional, and spiritual burden sustained by HCWs has been called burnout, moral distress, compassion fatigue, and a host of similar monikers, but there is one marker of HCW mental health that has received more empirical attention and has more psychometric validity than any other: emotional exhaustion (EE).

According to a psychometric meta-analysis, of the 3 subscales of the Maslach Burnout Inventory^7^ (EE, depersonalization, and personal accomplishment), EE consistently produces the largest and most reliable Cronbach α estimates.^8^ To date, the most thoroughly researched HCW mental health marker is EE. Other metrics, such as depression, anxiety, posttraumatic stress disorder, and moral injury, may have more dire consequences than EE (this isn’t yet known), but they have not yet been studied as comprehensively, for as long, or in as many HCWs.^9^

Interventions designed to reduce EE among HCWs are ongoing and have included coaching^10,11^; changes to scheduling, workflows, and workload^12^; positive rounding^13,14^; bite-sized well-being strategies^15,16,17,18^; and structured efforts to increase meaning among groups of HCWs.^19^ These interventions are generally associated with a change in EE of at least 5 points on a 100-point scale. Among physicians, the implementation of electronic health records (EHRs) across the country between 2011 and 2014 co-occurred with a 9-point increase in EE, from 38% to 47%.^20,21^ Relative to the taxing nature of transitioning to EHRs, the effect of a global pandemic has not yet been thoroughly studied.

Despite a rapid increase in research on HCW EE since 2010, few high-quality, large-scale longitudinal or cross-sectional studies are available (see Shanafelt et al^22^ or Aiken et al^23^ for exceptions). Emotional exhaustion rates among HCWs were already considered alarmingly high before the pandemic,^24,25^ prompting the 2019 release of the National Academy of Medicine consensus report^26^ on taking action against clinician burnout. Recent cross-sectional surveys suggest that EE increased among HCWs during the COVID-19 pandemic,^27^ but to date, no large-scale surveys of the same individuals before and after the onset of the pandemic have been published. Despite the variety of opportunities for HCWs to experience pandemic exhaustion, relatively little is known about the evolution of EE among HCWs over time.

## Methods

This survey study followed the American Association for Public Opinion Research (AAPOR) reporting guideline and was approved by the Duke University Health System’s institutional review board, which waived the need for consent owing to the confidential and deidentified nature of the data set. Emotional exhaustion was assessed using routine electronic (via email and/or access to a generic link) administration of the Safety, Communication, Organizational Reliability, Physician and Employee Burnout and Engagement (SCORE)^28^ survey in 2 US health care systems across 76 widely geographically dispersed hospitals before the pandemic (September 2019), after the start of the pandemic (September 2020), and after the introduction of vaccines and vaccine mandates and the rise of the Delta variant (September 2021 in the first system and December 2021 through January 2022 in the second system). All eligible staff at the 2 health systems with 50% or greater full-time equivalent commitment to a specific work setting for at least 4 consecutive weeks were asked to complete the survey. Demographic variables such as age, sex, and race were not collected during these routine survey administrations. The SCORE survey assesses safety culture and workforce well-being and engagement, including an EE scale and an emotional exhaustion climate (EEclim) scale, because HCW well-being was increasingly recognized as common,^24,25^ expensive,^29^ and treatable.^17,18^ Emotional exhaustion assesses the extent to which one feels drained, overwhelmed, and unable to meet demands. Example items from the SCORE survey include “I feel frustrated by my job” and “events in this work setting affect my life in an emotionally unhealthy way.” In parallel, EEclim assesses the extent to which one perceives EE in their colleagues. Example items include “people in this work setting feel frustrated by their jobs” and “events in this work setting affect the lives of people here in an emotionally unhealthy way.” The response scale for EE and EEclim range from 1 (strongly disagree) to 5 (strongly agree), such that higher scores reflect higher levels of exhaustion (eFigure in the Supplement). Years at the facility were assessed as less than 1 year, 1 to 10 years, or 11 years or more.

### Statistical Analysis

Using the Cronbach α, a rough gauge of psychometric reliability (ranging between 0 and 1, with values of 0.7 and higher indicating acceptable reliability), the 5-item EE and EEclim scales consistently yield an α above 0.90.^13,17,18,28^ Together, EE and EEclim provide assessments of exhaustion in oneself and exhaustion witnessed in one’s colleagues (eTables 1 and 2 in the Supplement). To assess clustering of responses within work settings, intraclass correlation coefficients (ICCs) were calculated, with .01 considered a small effect, .10 considered a medium affect, and .25 considered a large effect.^30^

Cronbach αs for EE and EEclim were calculated for each wave. Results for EE and EEclim were aggregated overall, by role, and by aggregated roles using the standard technique^7,13,16,17,28,31,32^ of percent exhausted (ie, by calculating the percentage of respondents reporting neutral or higher on the 5-point response scale), also known as “percent concerning.” Percent concerning for EE and EEclim are the percentage of respondents in a group reporting exhaustion levels in themselves (EE) and their work setting (EEclim) that are unfavorable. Means, SDs, and percentages (%EE for those reporting EE and %EEclim for those reporting EEclim) are reported. A generalized linear mixed effect model was used to assess changes in %EE and %EEclim from 2019 to 2020, from 2020 to 2021, and from 2019 to 2021-2022 by HCW role and system, where HCWs are nested within facilities and facilities are nested within systems. The model included fixed effects for time period, HCW role, and years at the facility and system and random effects for facilities nested within systems.

All hypothesis tests in the mixed model were conducted in SAS PROC GLIMMIX. A *P *value of <.05 was considered statistically significant. Because this was an exploration of descriptive data of exhaustion over 3 years, all tests were 2-sided. Statistical analyses were performed in SAS, version 9.4 (SAS Institute Inc).

## Results

Electronic surveys were returned by 37 187 (of 49 936) HCWs in 2019, by 38 460 (of 45 268) in 2020, and by 31 475 (of 41 224) in 2021 to 2022 for overall response rates of 74.5%, 85.0%, and 76.4%, respectively. The overall sample comprised 107 122 completed surveys. Nursing was the most frequently reported role (n = 43 918 [40.9%]). Overall, 17 786 respondents (16.9%) reported less than 1 year at their facility, 59 226 (56.2%) reported 1 to 10 years, and 28 337 (26.9%) reported 11 years or more (eTables 3 and 4 in the Supplement). Results were missing in 2019, 2020, and 2021-2022 for 0.7%, 0.7%, and 1.5% for EE and 0.7%, 0.7%, and 1.7% for EEclim, respectively. The numbers of respondents and percent concerning results for EE and EEclim are presented in Table 1. For EE, the Cronbach α was .93 for all 3 survey periods; for EEclim, the Cronbach α was .91 in 2019, .92 in 2020, and .92 in 2021 to 2022. The ICC for EE was 0.15 in 2019, 0.17 in 2020, and 0.17 in 2021 to 2022; for EEclim, it was 0.22 in 2019, 0.24 in 2020, and 0.24 in 2021 to 2022 (see the eAppendix in the Supplement for additional details on the scales).

**Table 1.** Number of Participants and Percent Concerning for Emotional Exhaustion and Emotional Exhaustion Climate Across Years^a^

Role^b^	No. of participants				Percent Concerning (95% CI)					
	2019 to 2021-2022	2019	2020	2021-2022	Emotional exhaustion			Emotional exhaustion climate		
					2019	2020	2021-2022	2019	2020	2021-2022
Overall	107 122	37 187	38 460	31 475	31.8 (30.0-33.7)	34.6 (32.5-36.8)	40.4 (38.1-42.8)	53.3 (52.8-53.8)	59.8 (59.3-60.3)	64.9 (64.3-65.5)
Physician										
Resident	846	216	323	307	24.6 (19.9-30.3)	24.7 (20.7-29.6)	34.4 (29.7-39.8)	44.5 (37.2-52.2)	43.9 (38.6-50.4)	57.5 (51.0-64.1)
Not employed by hospital	395	160	102	133	28.9 (23.0-36.2)	23.8 (17.1-33.2)	34.6 (27.7-43.3)	53.6 (45.0-62.3)	40.6 (30.6-52.9)	59.1 (49.7-68.8)
Attending or staff	3016	1280	1115	621	33.4 (30.6-36.5)	29.8 (27.0-32.9)	40.1 (36.2-44.4)	53.9 (50.8-56.8)	45.1 (41.8-47.8)	59.1 (54.3-63.4)
Administrator or manager	6800	2275	2443	2082	26.0 (23.8-28.3)	30.2 (27.9-32.6)	35.0 (32.4-37.8)	37.3 (35.3-39.4)	46.3 (44.3-48.7)	52.6 (50.3-54.8)
Administrative support (clerk, secretary, or receptionist)	5210	1770	1941	1499	29.0 (26.6-31.7)	31.3 (28.9-34.0)	38.0 (35.1-41.2)	46.9 (45.5-48.5)	53.5 (52.1-54.8)	57.5 (55.7-59.6)
Other	14 933	5555	6147	3231	31.0 (29.0-33.1)	35.8 (33.6-38.1)	38.0 (35.5-40.7)	48.1 (45.3-50.6)	59.6 (56.9-62.2)	61.1 (58.5-63.9)
Therapist (RT, PT, OT, or speech)	6282	2083	2142	2057	27.2 (25.0-29.6)	34.4 (31.9-37.1)	39.1 (36.4-42.1)	43.9 (41.7-46.2)	51.9 (49.6-54.1)	62.3 (60.0-64.6)
Clinical social worker	1247	462	470	315	34.1 (29.9-38.8)	37.4 (33.1-42.2)	39.6 (34.6-45.3)	46.6 (42.6-50.5)	55.8 (51.5-60.0)	62.9 (58.8-66.9)
Pharmacist	2048	642	692	714	33.5 (30.0-37.5)	36.4 (32.8-40.4)	41.9 (38.1-46.0)	48.2 (45.7-50.8)	54.9 (52.4-57.2)	62.6 (59.9-65.5)
Technician (eg, surgical, laboratory, EKG, radiology, or pharmacy	4938	1573	1719	1646	32.9 (30.2-35.9)	39.1 (36.3-42.2)	42.1 (39.1-45.4)	46.7 (44.9-48.9)	57.0 (54.8-58.8)	63.2 (61.1-65.5)
Technologist (eg, surgical, laboratory, or radiology)	8478	3026	2852	2600	31.6 (29.4-34.0)	38.1 (35.5-40.8)	42.8 (40.0-45.8)	59.9 (57.7-62.2)	68.1 (65.8-70.2)	67.7 (65.7-69.9)
Clinical support (medical assistant, EMT, etc)	1846	693	752	401	39.5 (35.5-43.9)	42.8 (39.0-47.0)	46.8 (41.5-52.7)	56.9 (52.8-61.1)	65.3 (61.2-69.2)	69.0 (63.6-74.4)
Nurses aide, CNA, PCA, PCT	7165	2437	2318	2410	37.6 (35.1-40.4)	42.5 (39.7-45.5)	46.6 (43.6-49.8)	60.7 (59.7-61.5)	66.9 (66.0-67.7)	69.9 (68.9-70.8)
Nurse	43 918	15 015	15 444	13 459	40.6 (38.4-42.9)	46.5 (44.0-49.1)	49.2 (46.5-51.9)	62.5 (57.0-67.8)	66.9 (61.5-71.6)	74.7 (69.6-80.4)

Abbreviations: CNA, certified nursing assistant; EKG, electrocardiography; EMT, emergency medical technician; OT, occupational therapist; PCA, patient care assistant; PCT, patient care technician; PT, physical therapist; RT, recreational therapist.

^a^ Model was adjusted for health care worker role, system, time period, and years at facility as fixed effects and for facilities nested within systems as random effects.

^b^ Health care worker roles are sorted from lowest to highest percentage reporting emotional exhaustion in 2021 to 2022.

From September 2019 to September 2021 through January 2022, overall %EE increased from 31.8% (95% CI, 30.0%-33.7%) to 34.6% (95% CI, 32.5%-36.8%) to 40.4% (95% CI, 38.1%-42.8%) with a proportional increase in %EE of 26.9% (95% CI, 22.2%-31.8%) (Table 2).

**Table 2.** Proportional Change by Years of Percent Concerning for Emotional Exhaustion and Emotional Exhaustion Climate Across Years^a^

Role^b^	% (95% CI)					
	Emotional exhaustion			Emotional exhaustion climate		
	2020 vs 2019^c^	2021 to 2022 vs 2020^d^	2021 to 2022 vs 2019^e^	2020 vs 2019^c^	2021 to 2022 vs 2020^d^	2021 to 2022 vs 2019^e^
Overall	8.7 (4.3 to 13.4)^f^	16.7 (12.1 to 21.5)^f^	26.9 (22.2 to 31.8)^f^	12.3 (10.8 to 13.8)^f^	8.4 (6.9 to 10.1)^f^	21.8 (20.0 to 23.6)^f^
Physician						
Resident	0.7 (−22.7 to 31.1)	39.3 (12.2 to 73.0)^f^	40.2 (10.0 to 78.7)^f^	−1.7 (−19.4 to 23.9)	30.4 (9.9 to 57.0)^f^	28.9 (7.5 to 58.3)^f^
Not employed by hospital	−17.6 (−44.5 to 22.4)	45.6 (−1.7 to 115.4)	20.0 (−11.9 to 63.4)	−24.5 (−46.4 to 4.3)	45.4 (9.3 to 107.4)^f^	9.9 (−14.3 to 40.9)
Attending or staff	−11.0 (−20.2 to −0.7)^f^	34.8 (19.4 to 52.2)^f^	20.0 (7.3 to 34.3)^f^	−16.3 (−23.9 to −9.6)^f^	31.5 (18.1 to 47.3)^f^	9.8 (−0.5 to 20.5)
Administrator or manager	16.1 (6.2 to 26.9)^f^	16.1 (7.0 to 25.8)^f^	34.7 (23.3 to 47.2)^f^	24.3 (14.5 to 34.3)^f^	13.3 (6.3 to 20.9)^f^	40.7 (30.6 to 51.2)^f^
Administrative support (clerk, secretary, or receptionist)	7.9 (−1.8 to 18.5)	21.3 (11.2 to 32.3)^f^	30.9 (19.2 to 43.8)^f^	14.0 (5.9 to 21.3)^f^	13.7 (6.8 to 21.6)^f^	29.8 (20.0 to 39.0)^f^
Other	15.6 (9.8 to 21.7)^f^	6.2 (0.6 to 12.2)^f^	22.8 (15.8 to 30.3)^f^	14.2 (9.0 to 19.1)^f^	7.8 (2.7 to 12.3)^f^	22.8 (16.0 to 28.5)^f^
Therapist (RT, PT, OT, or speech)	26.3 (16.0 to 37.5)^f^	13.8 (5.7 to 22.4)^f^	43.6 (32.3 to 55.9)^f^	18.3 (10.1 to 26.2)^f^	19.9 (13.9 to 27.5)^f^	42.2 (33.3 to 50.5)^f^
Clinical social worker	9.7 (−6.5 to 28.9)	6.0 (−10.2 to 25.0)	16.3 (−2.1 to 38.1)	7.1 (−3.9 to 19.0)	11.7 (0.6 to 23.9)^f^	19.6 (6.0 to 35.3)^f^
Pharmacist	8.7 (−4.9 to 24.2)	15.1 (2.2 to 29.6)^f^	25.1 (10.3 to 42.0)^f^	19.4 (6.4 to 34.0)^f^	12.7 (2.2 to 24.0)^f^	34.5 (21.2 to 50.2)^f^
Technician (eg, surgical, laboratory, EKG, radiology, or pharmacy	18.9 (9.1 to 29.7)^f^	7.7 (−0.1 to 16.1)	28.0 (17.5 to 39.5)^f^	24.1 (15.4 to 33.6)^f^	2.6 (−4.0 to 9.8)	27.1 (18.2 to 37.1)^f^
Technologist (eg, surgical, laboratory, or radiology)	20.4 (12.5 to 28.8)^f^	12.5 (5.8 to 19.6)^f^	35.3 (26.7 to 44.6)^f^	21.2 (14.4 to 27.7)^f^	11.4 (6.2 to 17.6)^f^	35.3 (28.3 to 43.0)^f^
Clinical support (medical assistant, EMT, etc)	8.5 (−3.6 to 22.0)	9.3 (−4.2 to 24.6)	18.5 (2.9 to 36.5)^f^	14.4 (4.3 to 26.6)^f^	5.9 (−4.3 to 16.9)	20.8 (7.7 to 35.3)^f^
Nurses aide, CNA, PCA, or PCT	13.0 (6.1 to 20.5)^f^	9.6 (3.4 to 16.2)^f^	23.9 (16.5 to 31.7)^f^	13.6 (8.3 to 19.7)^f^	−0.5 (−5.1 to 4.1)	13.0 (7.6 to 18.6)^f^
Nurse	14.5 (11.8 to 17.3)^f^	5.8 (3.4 to 8.2)^f^	21.1 (18.3 to 24.0)^f^	10.2 (8.1 to 12.4)^f^	4.5 (2.5 to 6.5)^f^	15.1 (12.9 to 17.5)^f^

Abbreviations: CNA, certified nursing assistant; EKG, electrocardiography; EMT, emergency medical technician; OT, occupational therapist; PCA, patient care assistant; PCT, patient care technician; PT, physical therapist; RT, recreational therapist.

^a^ Model was adjusted for health care worker role, system, time period, and years at facility as fixed effects and facilities nested within systems as random effects. A positive proportional change indicates that the later year had a higher level of reported emotional exhaustion or emotional exhaustion climate compared with the previous year; a negative proportional change indicates that the later year had a lower level compared with the previous year.

^b^ Health care worker roles are sorted from lowest to highest percentage reporting emotional exhaustion in 2021 to 2022.

^c^ Proportional change comparing 2020 with 2019: (2020 percentage − 2019 percentage) / 2019 percentage × 100%.

^d^ Proportional change comparing 2021 to 2022 with 2020: (2021-2022 percentage − 2020 percentage) / 2020 percentage × 100%.

^e^ Proportional change comparing 2021 to 2022 with 2019: (2021-2022 percentage − 2019 percentage) / 2019 percentage × 100%.

^f^ Significant at the .05 level.

**Figure 1.**
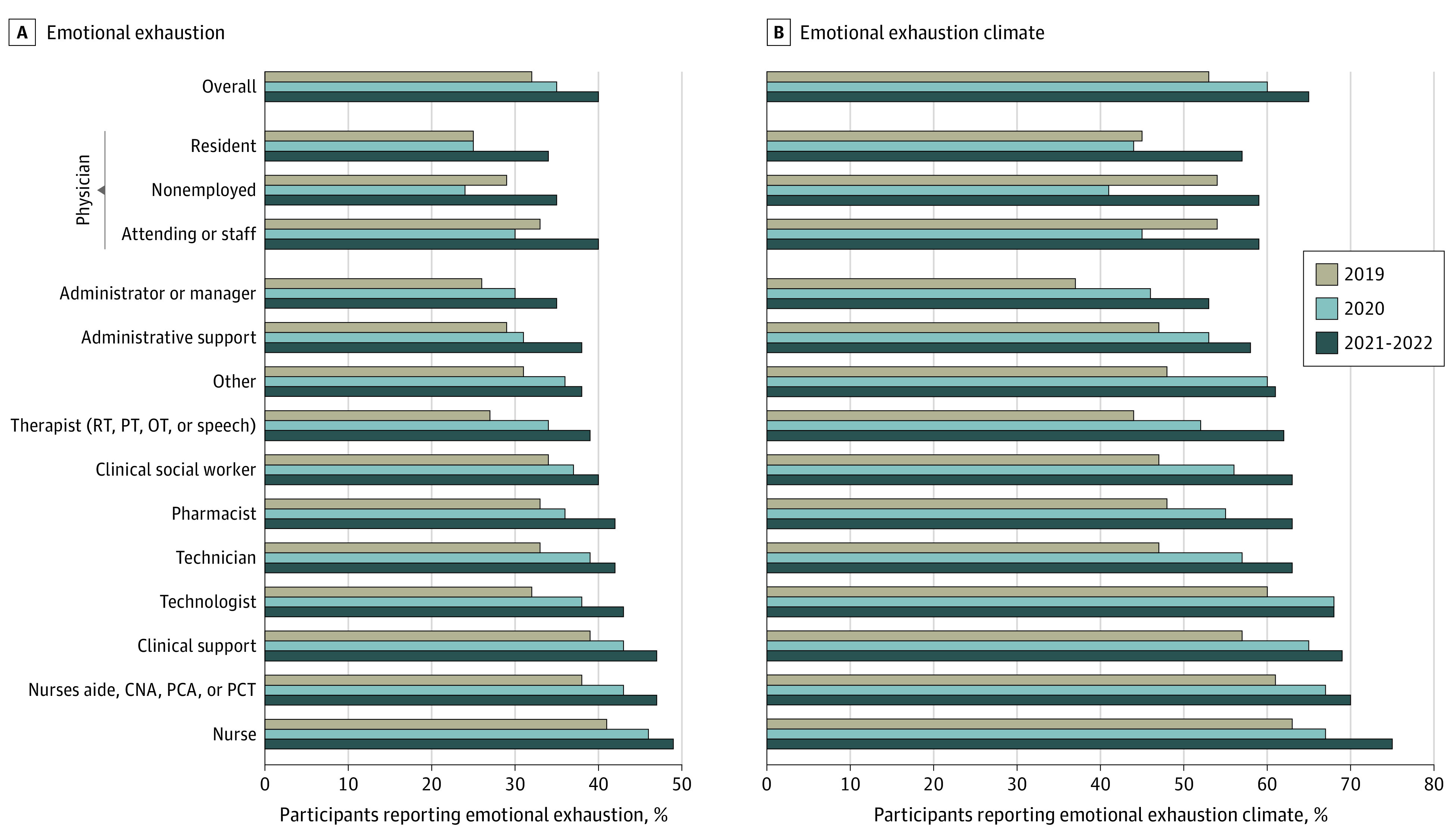
Reported Emotional Exhaustion and Emotional Exhaustion Climate by Health Care Worker Role CNA indicates certified nursing assistant; OT, occupational therapist; PCA, patient care assistant; PCT, patient care technician; PT, physical therapist; and RT, recreational therapist.

**Figure 2.**
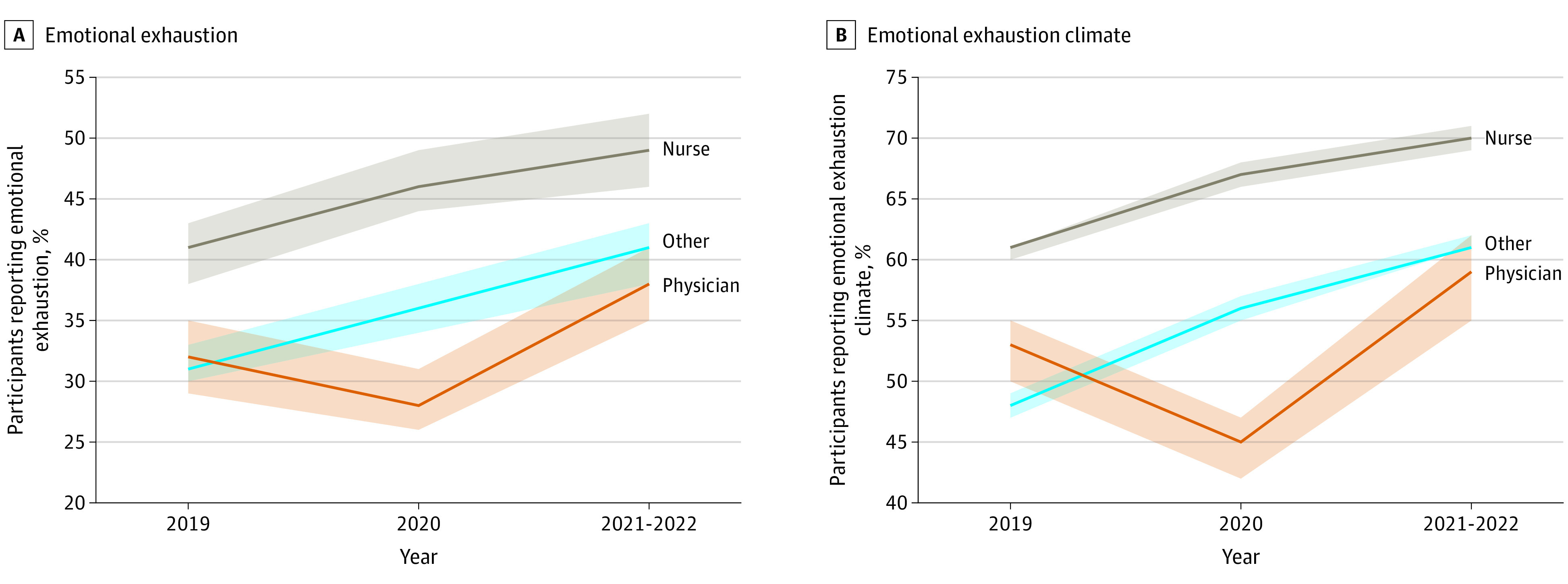
Reported Emotional Exhaustion and Emotional Exhaustion Climate by Aggregated Health Care Worker Role Shaded areas indicate 95% CIs.

Role-specific results by year are presented in Figure 1, and aggregated roles by year are presented in Figure 2. The aggregated categories of physicians reported a decrease in %EE from 31.8% (95% CI, 29.3%-34.5%) to 28.3% (95% CI, 25.9%-31.0%) but an increase during the second year to 37.8% (95% CI, 34.7%-41.3%). Conversely, nurses reported an increase in %EE during the first year from 40.6% (95% CI, 38.4%-42.9%) to 46.5% (95% CI, 44.0%-49.1%) and another increase during the second year to 49.2% (95% CI, 46.5%-51.9%). All other roles (except for physicians and nurses) were aggregated into a large “all others” category that showed an increase in %EE during the first year from 31.2% (95% CI, 29.7%-33.2%) to 36.3% (95% CI, 34.3%-38.3%) and another increase during the second year to 40.5% (95% CI, 38.3%-42.8%). Identical patterns of results were found for %EEclim in the aggregated HCW roles, and nearly identical patterns were seen for the specific role results (Tables 1 and 2). Both %EE and %EEclim increased from 2019 to 2021-2022 overall and by role, with the exception of physicians not employed by the hospital (Figure 1 and Table 1).

## Discussion

To date, this is the largest study of diverse HCW roles to demonstrate that exhaustion in 2021 to 2022 was higher than at the start of the pandemic. These HCWs reported that their personal EE increased and that they perceived EE increases in their colleagues. This outcome was the case for most HCW roles reporting their assessments in late 2019, late 2020, and late 2021 to early 2022. Every role, at every time point, reported higher emotional exhaustion in their colleagues than in themselves. Decades of research in social science demonstrate that people are unrealistically optimistic about their own health and well-being relative to that of others.^33^ Consequently, these HCW assessments of EE may be underreporting actual exhaustion, given the higher magnitude of EEclim reported. By late 2021, 2 of 3 HCWs reported witnessing EE in their colleagues (EEclim). The magnitude of the EE vs EEclim disconnect was more than 20 points on the 100-point scale, but the patterns of results were remarkably consistent across the 2 metrics of exhaustion. Intraclass correlation coefficient results indicated the extent to which HCWs from the same work setting resemble each other, demonstrating that 15% to 17% shared variance for EE and 22% to 24% for EEclim, considered medium and large clustering effects, respectively. These findings are preliminary evidence for a social contagion effect, whereby HCWs from the same work setting share an exhaustion norm that is considered to be nontrivial (eTable 2 in the Supplement).^30,34,35^

### Physician EE Relative to Other HCWs

The overall increase in %EE from 32% in 2019 to 40% in 2021 represented a proportional increase in EE of 27%. Relative to 2019, emotional exhaustion was higher by September 2020 for nurses, nurses aides, technologists, technicians, therapists, administrators or managers, and others. By 2021 to 2022, all HCW roles reported increases in EE and EEclim except for the smallest HCW role category, physicians not employed by the hospital. Despite the robust pattern of increasing exhaustion reported across HCW roles, physician respondents were unique. Their EE actually decreased from 2019 to 2020, then sharply increased the next year. This decrease in physician EE during 2020 result aligns with the findings of Shanafelt et al,^22^ who reported lower EE in 2020 in a national sample of physicians. Flexibilities afforded by increases in telehealth and decreases in patient volume may explain some of the 2020 decrease in physician EE. In a recent study to understand the association between pandemic phases and EHR usage, Tsai, Boakak, and Hinz^36^ examined total received messages and total time in the system for the same providers in 2019, 2020, and 2021. They reported significant decreases in EHR use in 2020 and significant increases in EHR use in 2021. These results line up with the physician exhaustion results reported here, showing a decrease in 2020 (when patient volumes and EHR use were lower) and an increase in 2021 (when patient volumes and EHR use were higher).

The decrease in physician EE and EEclim reported in 2020 was followed by a sharp increase in EE and EEclim by the end of 2021. During that year, EE increased by 9 points among residents, by 11 points among physicians not employed by the hospital, and by 10 points among attending and staff physicians. These are the largest single-year increases reported by any roles across any time frame, exceeding what was found during a 3-year period when EHRs were introduced nationally.^20,21^ The decreases in physician EE in 2020 were completely undone by 2021, and moreover, the 2021 EE levels were substantially worse. Exhaustion levels varied considerably by HCW role within each of the 3 years, so the vulnerability of a given HCW to increases in EE was a function of both role and year of assessment.

Differences between physician and nonphysician assessments of workplace norms are not new,^37^ and the emotional exhaustion discrepancies reported here for September 2020 are similar to previous results from April 2020, when nurses reported significantly more acute stress, depression, and anxiety than their physician counterparts.^38^ Anecdotally, nurses in 2020 reported higher patient volumes, rapidly evolving processes and standards, and significant burdens from child care, remote learning, and work-life integration concerns. Initiation of well-being interventions by busy and exhausted HCWs is made more challenging when EE increases, because taking time to do something about well-being then becomes one more thing on an overwhelmed to-do list.

Before the pandemic, many institutions considered themselves progressive if they had convened a task force to examine HCW burnout. We now know that HCW burnout is a parallel pandemic that will be felt for many years to come. Leaders need tiered options for responding to burnout, but the evidence has not kept pace with the demand for well-being resources in health care settings. The National Academy of Medicine^26^ report on clinician burnout and the Surgeon General’s advisory on HCW burnout^39^ are calls to action for improving our health care delivery systems in ways that reduce EE and promote quality. We suggest that there is a need for both institutional^13,28,40^ and individual^16,17,18^ resources for HCW well-being, as evidenced by the role-specific findings reported here. Ideally, organizations would realize that fixing systems intrinsically includes a focus on individual HCW well-being through the application of meaningful and evidence-based options to improve it. We know this is possible because randomized controlled trial results^10,15^ demonstrate that we can cause EE among HCWs to decrease in magnitudes similar to the rates of increase reported here. Well-being resources for HCWs need to be broadly accessible and evidence-based, and their use should be role-modeled by leaders. We are only beginning to understand the toll of the pandemic on HCW well-being, and much more will be revealed over the next few years.

### Limitations

Results should be viewed in light of study design. Only EE and EEclim were assessed, so other dimensions of well-being, such as anxiety, depression, posttraumatic stress disorder, moral distress, and depersonalization were not included. We did not have access to physician specialty, work setting type, or other demographic or confounding variables. In 2020, some physician specialties were especially burdened in caring for patients with COVID-19 while others initially had decreased patient loads when routine procedures stopped or telehealth took hold. We did not have access to physician specialties to distinguish between these specialty groups. Also, these results came from 2 large US health systems, and although they are systems that are widely dispersed around the US, the extent to which they are broadly generalizable is unknown. Another limitation is the lack of unique identifiers that would allow for comparisons of the same HCWs with themselves from before to after the start of the pandemic, rather than the group-level differences reported here. Singling out respiratory therapists from occupational and physical therapists would have allowed for more specificity within that role but was not possible owing to the broad categories used. This study is also limited in its use of self-report data, which are at risk for response, selection, and social desirability biases. The good psychometric results for EE and EEclim within each year, as well as the large sample sizes and high response rates, help to buffer against some of these limitations.

## Conclusions

The findings of this survey study provide substantial evidence that EE has increased overall and in most HCW roles since the start of the COVID-19 pandemic. On a hopeful note, the magnitude of the increase in EE is within striking distance of the decreases in EE achieved in clinical trials of bite-sized interventions used by HCWs. We found that nurses, in particular, have shouldered more than their share of this pandemic since the start, and that physicians experienced a temporary reprieve in 2020 that was followed by the biggest single-year increase in EE for any role at any time point. Existing programs and resources to facilitate HCW well-being were inadequate before the pandemic and now appear to be woefully inadequate. This situation is made more complicated by the results reported here, because the workforce has even less reserve and capacity to initiate, sustain, and complete interventions to improve their well-being.

## Notes

Supplement. **eAppendix.** Background on the 5 Item Emotional Exhaustion and Emotional Exhaustion Climate Scales **eTable 1. **Chronbach’s Alphas for Emotional Exhaustion/Emotional Exhaustion Climate **eTable 2. **ICC **eFigure. **SCORE Survey Items **eTable 3. **Percent Concerning of Emotional Exhaustion by Health Care Worker Role and Years at Facility **eTable 4. **Percent Concerning of Emotional Exhaustion Climate by Health Care Worker Role and Years at Facility
eReferences

## References

[1] Litz BT, Stein N, Delaney E, . Moral injury and moral repair in war veterans: a preliminary model and intervention strategy. Clin Psychol Rev. 2009;29(8):695-706. doi:10.1016/j.cpr.2009.07.003 19683376

[2] Wang Z, Harold KG, Tong Y. Moral injury in Chinese health professionals during the COVID-19 pandemic. Psychol Trauma. 2022;14(2):250-257. doi:10.1037/tra0001026 34043381

[3] Mantri S, Lawson JM, Wang Z, Koenig HG. Identifying moral injury in healthcare professionals: The Moral Injury Symptom Scale-HP. J Relig Health. Published online July 17, 2020. doi:10.1007/s10943-020-01065-w PMC7366883 32681398

[4] Abbasi J. Pushed to their limits, 1 in 5 physicians intends to leave practice. JAMA. 2022;327(15):1435-1437. doi:10.1001/jama.2022.5074 35353134

[5] Li Y, Scherer N, Felix L, Kuper H. Prevalence of depression, anxiety and post-traumatic stress disorder in health care workers during the COVID-19 pandemic: A systematic review and meta-analysis. PLoS One. 2021;16(3):e0246454. doi:10.1371/journal.pone.0246454 33690641 PMC7946321

[6] Nieuwsma JA, O’Brien EC, Xu H, Smigelsky MA, Meador KG; VISN 6 MIRECC Workgroup; HERO Research Program. Patterns of potential moral injury in post-9/11 combat veterans and COVID-19 healthcare workers. J Gen Intern Med. 2022;37(8):2033-2040. doi:10.1007/s11606-022-07487-4 35381899 PMC8982664

[7] Maslach C, Jackson S. Maslach Burnout Inventory. Consulting Psychologists Press, Inc; 1981.

[8] Wheeler DL, Vassar M, Worley JA, Barnes LLB. A reliability generalization meta-analysis of coefficient alpha for the Maslach Burnout Inventory. Educ Psychol Meas. 2011;71(1):231-244. doi:10.1177/0013164410391579

[9] Rehder K, Adair KC, Sexton JB. The science of health care worker burnout: assessing and improving health care worker well-being. Arch Pathol Lab Med. 2021;145(9):1095-1109. doi:10.5858/arpa.2020-0557-RA 34459858

[10] Dyrbye LN, Shanafelt TD, Gill PR, Satele DV, West CP. Effect of a professional coaching intervention on the well-being and distress of physicians: a pilot randomized clinical trial. JAMA Intern Med. 2019;179(10):1406-1414. doi:10.1001/jamainternmed.2019.2425 31380892 PMC6686971

[11] Fainstad T, Mann A, Suresh K, . Effect of a novel online group-coaching program to reduce burnout in female resident physicians: a randomized clinical trial. JAMA Netw Open. 2022;5(5):e2210752. doi:10.1001/jamanetworkopen.2022.10752 35522281 PMC9077483

[12] Panagioti M, Panagopoulou E, Bower P, . Controlled interventions to reduce burnout in physicians: a systematic review and meta-analysis. JAMA Intern Med. 2017;177(2):195-205. doi:10.1001/jamainternmed.2016.7674 27918798

[13] Sexton JB, Adair KC, Profit J, . Safety culture and workforce well-being associations with positive leadership walkrounds. Jt Comm J Qual Patient Saf. 2021;47(7):403-411. doi:10.1016/j.jcjq.2021.04.001 34024756 PMC8240670

[14] Adair KC, Heath A, Frye M, . The Psychological Safety Scale of the Safety, Communication, Operational, Reliability, and Engagement (SCORE) Survey: a brief, diagnostic, and actionable metric for the ability to speak up in healthcare settings. J Patient Saf. 2022;18(6):513-520. doi:10.1097/PTS.0000000000001048 35985041 PMC9422763

[15] Profit J, Adair KC, Cui X, . Randomized controlled trial of the “WISER” intervention to reduce healthcare worker burnout. J Perinatol. 2021;41(9):2225-2234. doi:10.1038/s41372-021-01100-y 34366432 PMC8440181

[16] Adair KC, Rodriguez-Homs LG, Masoud S, Mosca PJ, Sexton JB. Gratitude at work: prospective cohort study of a web-based, single-exposure well-being intervention for health care workers. J Med internet Res. 2020;22(5):e15562. doi:10.2196/15562 32406864 PMC7256751

[17] Sexton JB, Adair KC. Forty-five good things: a prospective pilot study of the Three Good Things well-being intervention in the USA for healthcare worker emotional exhaustion, depression, work-life balance and happiness. BMJ Open. 2019;9(3):e022695. doi:10.1136/bmjopen-2018-022695 30898795 PMC6475256

[18] Adair KC, Kennedy LA, Sexton JB. Three Good Tools: Positively reflecting backwards and forwards is associated with robust improvements in well-being across three distinct interventions. J Posit Psychol. Published online July 9, 2020. doi:10.1080/17439760.2020.1789707 PMC8294345 34295357

[19] West CP, Dyrbye LN, Rabatin JT, . Intervention to promote physician well-being, job satisfaction, and professionalism: a randomized clinical trial. JAMA Intern Med. 2014;174(4):527-533. doi:10.1001/jamainternmed.2013.14387 24515493

[20] Shanafelt TD, Hasan O, Dyrbye LN, . Changes in burnout and satisfaction with work-life balance in physicians and the general US working population between 2011 and 2014. Mayo Clin Proc. 2015;90(12):1600-1613. doi:10.1016/j.mayocp.2015.08.023 26653297

[21] Shanafelt TD. Physician Well-being 2.0: where are we and where are we going? Mayo Clin Proc. 2021;96(10):2682-2693. doi:10.1016/j.mayocp.2021.06.005 34607637

[22] Shanafelt TD, West CP, Sinsky C, . Changes in burnout and satisfaction with work-life integration in physicians and the general US working population between 2011 and 2020. Mayo Clin Proc. 2022;97(3):491-506. doi:10.1016/j.mayocp.2021.11.021 35246286

[23] Aiken LH, Sermeus W, Van den Heede K, . Patient safety, satisfaction, and quality of hospital care: cross sectional surveys of nurses and patients in 12 countries in Europe and the United States. BMJ. 2012;344:e1717. doi:10.1136/bmj.e1717 22434089 PMC3308724

[24] Poghosyan L, Clarke SP, Finlayson M, Aiken LH. Nurse burnout and quality of care: cross-national investigation in six countries. Res Nurs Health. 2010;33(4):288-298. doi:10.1002/nur.20383 20645421 PMC2908908

[25] Shanafelt TD, West CP, Sinsky C, . Changes in burnout and satisfaction with work-life integration in physicians and the general US working population between 2011 and 2017. Mayo Clin Proc. 2019;94(9):1681-1694. doi:10.1016/j.mayocp.2018.10.023 30803733

[26] National Academies of Sciences, Engineering, and Medicine. Taking action against clinician burnout: a systems approach to professional well-being. National Academies Press; 2019. 31940160

[27] Haidari E, Main E, Cui X, . Maternal and neonatal health care worker well-being and patient safety climate amid the COVID-19 pandemic. J Perinatol. 2021;41:961-969. doi:10.1038/s41372-021-01014-9 33727700 PMC7962434

[28] Sexton JB, Adair KC, Leonard MW, . Providing feedback following Leadership WalkRounds is associated with better patient safety culture, higher employee engagement and lower burnout. BMJ Qual Saf. 2018;27(4):261-270. doi:10.1136/bmjqs-2016-006399 28993441 PMC5867443

[29] Han S, Shanafelt TD, Sinsky CA, . Estimating the attributable cost of physician burnout in the United States. Ann Intern Med. 2019;170(11):784-790. doi:10.7326/M18-1422 31132791

[30] LeBreton JM, Senter JL. Answers to 20 questions about interrater reliability and interrater agreement. Organ Res Methods. 2008;11(4):815-852. doi:10.1177/1094428106296642

[31] Schwartz SP, Adair KC, Bae J, . Work-life balance behaviours cluster in work settings and relate to burnout and safety culture: a cross-sectional survey analysis. BMJ Qual Saf. 2019;28(2):142-150. doi:10.1136/bmjqs-2018-007933 30309912 PMC6365921

[32] Rehder KJ, Adair KC, Hadley A, . Associations between a new disruptive behaviors scale and teamwork, patient safety, work-life balance, burnout, and depression. Jt Comm J Qual Patient Saf. 2020;46(1):18-26. doi:10.1016/j.jcjq.2019.09.004 31706686

[33] Dunning D, Heath C, Suls JM. Flawed self-assessment: implications for health, education, and the workplace. Psychol Sci Public Interest. 2004;5(3):69-106. doi:10.1111/j.1529-1006.2004.00018.x 26158995

[34] Smits M, Wagner C, Spreeuwenberg P, van der Wal G, Groenewegen PP. Measuring patient safety culture: an assessment of the clustering of responses at unit level and hospital level. Qual Saf Health Care. 2009;18(4):292-296. doi:10.1136/qshc.2007.025965 19651934

[35] Bliese PD. Within-group agreement, non-independence, and reliability: Implications for data aggregation and analysis. In: Multilevel Theory, Research, and Methods in Organizations: Foundations, Extensions, and New Directions. Jossey-Bass; 2000:349-381.

[36] Tsai T, Boazak M, Hinz ERM. Increased clinician time using electronic health records during COVID-19 pandemic. AMIA Annu Symp Proc. 2022;2021:1159-1168. 35308951 PMC8861678

[37] Sexton JB, Thomas EJ, Helmreich RL. Error, stress, and teamwork in medicine and aviation: cross sectional surveys. BMJ. 2000;320(7237):745-749. doi:10.1136/bmj.320.7237.745 10720356 PMC27316

[38] Shechter A, Diaz F, Moise N, . Psychological distress, coping behaviors, and preferences for support among New York healthcare workers during the COVID-19 pandemic. Gen Hosp Psychiatry. 2020;66:1-8. doi:10.1016/j.genhosppsych.2020.06.007 32590254 PMC7297159

[39] US Department of Health and Human Services. Health worker burnout. 2022. Accessed August 22, 2022. https://www.hhs.gov/surgeongeneral/priorities/health-worker-burnout/index.html

[40] Sexton JB, Adair KC, Profit J, . Perceptions of Institutional Support for “Second Victims” Are Associated with Safety Culture and Workforce Well-Being. Jt Comm J Qual Patient Saf. 2021;47(5):306-312. doi:10.1016/j.jcjq.2020.12.001 33563556

