# Supplementary material for: Emotional Exhaustion Among US Health Care Workers Before and During the COVID-19 Pandemic, 2019-2021
Source: JAMA Netw Open. 2022 Sep 21;5(9):e2232748. doi: 10.1001/jamanetworkopen.2022.32748 (PMC9494188; doi:10.1001/jamanetworkopen.2022.32748)
Supplement: Supplement. — eAppendix. Background on the 5 Item Emotional Exhaustion and Emotional Exhaustion Climate Scales eTable 1. Chronbach’s Alphas for Emotional Exhaustion/Emotional Exhaustion Climate eTable 2. ICC eFigure. SCORE Survey Items eTable 3. Percent Concerning of Emotional Exhaustion by Health Care Worker Role and Years at Facility eTable 4. Percent Concerning of Emotional Exhaustion Climate by Health Care Worker Role and Years at Facility eReferences [file jamanetwopen-e2232748-s001.pdf]

Supplemental Online Content

Sexton JB, Adair KC, Proulx J, et al. Emotional exhaustion among US health care workers before and during the COVID-19 pandemic, 2019-2021. *JAMA Netw Open*. 2022;5(9):e2232748. doi:10.1001/jamanetworkopen.2022.32748

eAppendix. Background on the 5 Item Emotional Exhaustion and Emotional Exhaustion Climate Scales
eTable 1. Chronbach's Alphas for Emotional Exhaustion/Emotional Exhaustion Climate
eTable 2. ICC
eFigure 1. SCORE Survey Items
eTable 3. Percent Concerning of Emotional Exhaustion by Health Care Worker Role and Years at Facility
eTable 4. Percent Concerning of Emotional Exhaustion Climate by Health Care Worker Role and Years at Facility
eReferences

This supplemental material has been provided by the authors to give readers additional information about their work.

eAppendix. Background on the 5 Item Emotional Exhaustion and Emotional Exhaustion Climate Scales

The Maslach Burnout Inventory is the gold standard for assessing burnout and has been used extensively with healthcare workers. A meta-analysis has revealed that of the three sub-scales (emotional exhaustion, depersonalization, and personal accomplishment), emotional exhaustion consistently produces the largest and most reliable coefficient alpha estimates, while depersonalization and personal accomplishment were both lower and less dependable.[1] Emotional exhaustion is the most widely studied and reported domain, and assesses the extent to which one feels emotionally overextended and depleted by work. In addition to being more psychometrically robust, emotional exhaustion can be used to discriminate between burned out and non-burned out outpatients suffering from work-related neurasthenia (according to ICD-10 criteria).[2] We use a 5-item derivative[3] of the original 9-item emotional exhaustion scale.[2] Having used and published this short version of the emotional exhaustion scale[4–10] we know from numerous large samples that it holds up psychometrically and is responsive to interventions. Correspondingly, Emotional Exhaustion Climate provides assessments of exhaustion in one's colleagues.

Both exhaustion metrics have excellent reliability as demonstrated through Cronbach's alpha, used to measure internal reliability, or how closely related the set of questions are as a group. Alphas range from 0 to 1, with a reliability coefficient of at least .70 being acceptable for early-stage research, .80 for implementing cutoff scores, and .90 if clinically important decisions are being made.[11,12]
In the current study, Cronbach's alphas for emotional exhaustion and emotional exhaustion climate were excellent and are reported by healthcare worker role and year (eTable 1).

eTable1. Chronbach's Alphas for Emotional Exhaustion/Emotional Exhaustion Climate

	2019	2020	2021/ 2022
Admin Support (Clerk/Secretary/Receptionist)	.92/.91	.93/.92	.94/.92
Administrator/Manager	.92/.89	.92/.90	.93/.91
Clinical Social Worker	.92/.89	.93/.90	.93/.90
Clinical Support (Medical Assistant/EMT/etc.)	.93/.89	.92/.90	.92/.90
Nurse	.93/.92	.94/.92	.94/.93
Nurses Aide/CNA/PCA/PCT	.93/.90	.92/.90	.93/.90
Other	.92/.90	.93/.91	.93/.91
Pharmacist	.93/.92	.94/.92	.94/.93
Physician: Attending/Staff	.93/.92	.93/.91	.93/.93

Physicians not employed by the hospital	.92/.93	.89/.91	.93/.94
Physician: Resident	.94/.91	.91/.91	.93/.93
Technician (e.g., Surg/Lab/EKG/Rad/Pharm)	.94/.92	.93/.92	.94/.92
Technologist (e.g. Surg./Lab/Rad)	.92/.91	.93/.92	.93/.92
Therapist (RT/PT/OT/Speech)	.92/.91	.93/.91	.93/.91
Overall	.93/.91	.93/.92	.93/.92

The overall α's for EE and EEclim were remarkably similar to results in another large sample of HCWs (n=16,797) where Cronbach's alphas were .92 and .90, respectively.[3] These two exhaustion metrics consistently demonstrate high reliability.

In addition to α's assessing how closely related a set of questions are as a group, intraclass correlations can be used to show correlations among observations within a cluster. In the current study, the intraclass correlation coefficient (ICC) estimates the extent to which HCWs within a given work setting resemble each other. In other words, the ICCs describe how strongly healthcare workers *in the same work setting resemble each other* in their assessments of EE and EEclim. Across a number of organizational climate studies, James (1982) reported a median ICC of .12 for measures of organizational climate, suggesting a typical shared group norm of 12%.[13] In the current study, this was assessed using an ICC model adjusted for healthcare worker role and years at facility as fixed effects and work setting as a random effect (eTable 2).

**eTable 2. ICC**			
	**2019**	**2020**	**2021/2022**
EE	0.153	0.173	0.174
EE Climate	0.221	0.238	0.236

Note: EE and EEclim were used as continuous variables (0-100 point).

This model demonstrated considerable shared variance for emotional exhaustion and emotional exhaustion climate scales *within work settings*. On average, the amount of shared variance at the work setting level in assessments of emotional exhaustion was 15-17%, and emotional exhaustion climate was 22-24%. These ICCs are similar to previously reported results on a statewide sample from Michigan.[3] Both scales and response options are provided in eFigure 1. The shared perceptions of EE suggest that emotional exhaustion is not only an individual difference between HCWs, but also acts like a group norm. At least in part, the extent to which one reports emotional exhaustion appears to depend upon the HCWs with whom one works (in the same work setting). In particular, EEclim ICCs suggest that between a fifth and a fourth this exhaustion metric can be attributed to the work setting of the respondent. In essence, this large

© 2022 Sexton JB et al. *JAMA Network Open.*

sample of HCWs demonstrates that EE and EEclim perform remarkably similar to traditional metrics of safety culture and organizational climate, indicating a shared experience of a group norm.[14–17] This is preliminary evidence for a social contagion effect of HCW exhaustion.

Statistical analysis details of Table 2.

Based on our generalized linear mixed effect model we first estimated the percent of respondents reporting emotional exhaustion (%EE) and a climate of emotional exhaustion (%EEclim) in 2019, 2020, and 2021 (Table 1). In order to demonstrate how much %EE and %EEclim changed from one year to another, we additionally estimated the proportional change of %EE and %EEclim across years adjusting for the same covariates. For example, proportional change comparing 2019 with 2020 was calculated as: (percent concerning in 2020 - percent concerning in 2019)/percent concerning in 2019*100%. 95%CI not including 0 indicates that there was a significant change from one year to another, which were highlighted in bold in Table 2. Details of the calculation were also included in the footnotes of Table 2.

Proportional Increases in EE and EEclim Results

Overall, %EE increased significantly in the first year of the pandemic (2019 to 2020) from 32% (95%CI: 30, 34) to 35% (95%CI: 33, 37); with a proportional increase in EE of 8.3% (95%CI: 4.3, 13.4; Table 2). In other words, %EE increased significantly, over 8% in the first year. In the second year of the pandemic (2020 to 2021), %EE increased again from 35% (95%CI: 33, 37) to 40% (95%CI: 38, 43), with a proportional increase in %EE of 16.7% (95%CI: 12.1, 21.5; Table 2). In other words, the second year of the pandemic was associated with a 16% increase in %EE, which was twice as big as the increase the previous year. During the 2 year period from 2019 to 2021, overall %EE increased from 32% (95%CI: 30, 34) to 40% (95%CI: 38, 43), with a proportional increase in %EE of 26.8% (95%CI: 22.2, 31.8; Table 2). During this period, the percent of HCWs reporting EE increased by 27%. This same pattern and explanation can be applied to the %EEclim results in Table 2.

© 2022 Sexton JB et al. *JAMA Network Open.*

eFigure1. SCORE Survey Items

Choose your responses using the scale below:

A	B	C	D	E	X
**Disagree Strongly**	**Disagree Slightly**	**Neutral**	**Agree Slightly**	**Agree Strongly**	**Not Applicable**

EMOTIONAL EXHAUSTION						
Events in this work setting affect my life in an emotionally unhealthy way.	A	B	C	D	E	X
I feel burned out from my work.	A	B	C	D	E	X
I feel fatigued when I get up in the morning and have to face another day on the job.	A	B	C	D	E	X
I feel frustrated by my job.	A	B	C	D	E	X
I feel I am working too hard on my job.	A	B	C	D	E	X

EMOTIONAL EXHAUSTION CLIMATE						
Events in this work setting affect the lives of people here in an emotionally unhealthy way.	A	B	C	D	E	X
People in this work setting are burned out from their work.	A	B	C	D	E	X
People in this work setting are fatigued from their work.	A	B	C	D	E	X
People in this work setting are frustrated by their jobs.	A	B	C	D	E	X
People in this work setting are working too hard on their jobs.	A	B	C	D	E	X

Averages near:
3 ("C" or Neutral) considered mild exhaustion
4 ("D" or Agree Slightly) considered moderate exhaustion
5 ("E" or Agree Strongly) considered severe exhaustion

eTable 3. Percent Concerning of Emotional Exhaustion by Health Care Worker Role and Years at Facility

	2019 to 2021/2022				2019				2020				2021/2022		
	Less than 1 year	1-10 years	11 years or more		Less than 1 year	1-10 years	11 years or more		Less than 1 year	1-10 years	11 years or more		Less than 1 year	1-10 years	11 years or more
Admin Support (Clerk/Secretary/Receptionist)	27.1	37.0	34.9		22.7	32.8	29.4		29.3	36.2	33.6		30.1	43.6	42.3
Administrator/Manager	25.7	33.0	30.6		22.5	27.9	25.8		25.9	33.8	31.3		28.6	37.7	35.3
Clinical Social Worker	31.7	42.1	39.0		30.7	37.3	40.2		31.6	44.4	33.8		33.3	45.2	45.1
Clinical Support (Medical Assistant/EMT/etc.)	30.0	46.9	52.2		27.7	42.1	50.5		31.4	49.5	56.8		31.9	50.5	48.7
Nurse	43.1	52.5	44.9		37.1	46.9	41.2		46.1	54.4	45.4		45.8	56.6	48.8
Nurses Aide/CNA/PCA/PCT	36.2	48.7	49.3		29.0	44.0	46.5		38.4	49.2	50.1		40.6	53.4	51.5
Other	27.1	39.1	37.1		23.6	33.8	34.6		29.6	41.9	36.6		28.8	42.5	42.3
Pharmacist	29.9	41.2	46.2		28.4	34.9	46.1		26.1	41.6	44.3		34.3	46.4	48.1
Physician: Attending/Staff	29.2	40.2	37.2		22.9	39.2	41.5		28.3	36.9	28.7		45.7	47.8	40.6
Physician: Not employed by hospital	15.0	34.3	34.0		0.0	30.2	38.0		0.0	23.4	33.3		100.0	45.5	28.3
Physician: Resident	34.0	34.4	NA		24.4	34.9	NA		32.3	27.9	NA		42.4	41.3	NA
Technician (e.g., Surg/Lab/ EKG/Rad/Pharm)	32.5	42.2	44.5		30.9	34.5	37.7		33.2	45.2	45.0		33.3	46.5	50.4
Technologist (e.g. Surg/ Lab/Rad.)	28.0	40.2	43.3		20.6	33.1	39.1		30.9	41.4	44.2		33.0	47.3	47.5
Therapist (RT/PT/OT/Speech)	26.6	37.8	38.7		20.5	29.4	33.5		30.7	38.9	39.3		28.1	45.6	43.3
**Overall**	35.2	45.5	41.1		29.6	39.9	37.7		37.1	46.8	41.0		38.9	50.5	45.6
**Number of participants by years at facility**	17786	59226	28337		6011	20373	10151		5557	22159	10090		6218	16694	8096

© 2022 Sexton JB et al. *JAMA Network Open.*

**eTable 4. Percent Concerning of Emotional Exhaustion Climate by Health Care Worker Role and Years at Facility**												
	**2019 to 2021/2022**			**2019**			**2020**			**2021/2022**		
	Less than 1 year	1-10 years	11 years or more	Less than 1 year	1-10 years	11 years or more	Less than 1 year	1-10 years	11 years or more	Less than 1 year	1-10 years	11 years or more
Admin Support (Clerk/Secretary/Receptionist)	46.3	56.3	56.7	40.1	49.4	53.7	48.2	57.4	55.8	52.0	63.7	61.1
Administrator/Manager	45.4	46.5	43.5	40.2	39.3	35.8	45.0	47.1	46.4	50.7	53.4	48.9
Clinical Social Worker	52.4	67.4	61.0	50.0	61.3	61.0	49.4	68.0	57.5	60.0	75.4	66.7
Clinical Support (Medical Assistant/EMT/etc.)	52.5	63.7	69.7	48.1	58.5	65.7	54.9	66.5	69.8	56.2	67.7	75.0
Nurse	61.2	68.3	60.4	58.1	63.2	57.4	61.5	69.6	61.2	63.7	72.6	63.0
Nurses Aide/CNA/PCA/PCT	57.3	66.9	68.2	51.3	61.6	66.0	60.7	68.3	72.0	59.9	71.3	66.3
Other	44.7	57.2	55.2	41.3	52.6	52.6	45.6	58.9	55.0	48.4	62.0	60.0
Pharmacist	45.6	57.3	59.1	40.7	46.5	55.3	43.0	59.9	58.8	51.5	64.6	63.1
Physician: Attending/Staff	42.4	52.5	52.9	40.0	52.6	57.5	40.3	47.5	45.2	53.3	61.8	54.0
Physician: Not employed by hospital	30.0	47.6	53.8	25.0	44.4	55.9	11.1	42.6	44.4	100.0	53.8	57.4
Physician: Resident	45.9	47.9	NA	37.9	45.7	NA	44.9	42.6	NA	52.4	55.2	NA
Technician (e.g., Surg/Lab/ EKG/Rad/Pharm)	48.6	57.2	59.4	46.0	48.4	50.5	53.9	59.9	62.9	47.2	63.2	64.1
Technologist (e.g. Surg/ Lab/Rad.)	43.5	55.9	58.0	34.9	48.5	52.3	43.8	57.3	59.2	51.6	63.1	63.4
Therapist (RT/PT/OT/Speech)	46.5	54.0	53.9	38.2	46.0	46.3	45.1	53.5	54.5	54.5	63.2	61.0
**Overall**	53.4	61.7	57.1	48.6	56.0	53.5	53.8	62.7	57.7	57.6	67.4	61.1
**Number of participants by years at facility**	17786	59226	28337	6011	20373	10151	5557	22159	10090	6218	16694	8096

© 2022 Sexton JB et al. *JAMA Network Open.*

eReferences

1. Loera B, Converso D, Viotti S. Evaluating the Psychometric Properties of the Maslach Burnout Inventory-Human Services Survey (MBI-HSS) among Italian Nurses: How Many Factors Must a Researcher Consider? *PLOS ONE*. 2014;9(12):e114987. doi:10.1371/journal.pone.0114987

2. Schaufeli WB, Bakker AB, Hoogduin K, Schaap C, Kladler A. On the clinical validity of the maslach burnout inventory and the burnout measure. *Psychol Health*. 2001;16(5):565-582. doi:10.1080/08870440108405527

3. Sexton JB, Adair KC, Leonard MW, et al. Providing feedback following Leadership WalkRounds is associated with better patient safety culture, higher employee engagement and lower burnout. *BMJ Qual Saf*. Published online October 9, 2017:bmjqs-2016-006399. doi:10.1136/bmjqs-2016-006399

4. Sexton JB, Schwartz SP, Chadwick WA, et al. The associations between work–life balance behaviours, teamwork climate and safety climate: cross-sectional survey introducing the work–life climate scale, psychometric properties, benchmarking data and future directions. *BMJ Qual Saf*. 2017;26(8):632-640. doi:10.1136/bmjqs-2016-006032

5. Profit J, Sharek PJ, Amspoker AB, et al. Burnout in the NICU setting and its relation to safety culture. *BMJ Qual Saf*. 2014;23(10):806-813. doi:10.1136/bmjqs-2014-002831

6. Schwartz SP, Adair KC, Bae J, et al. Work-life balance behaviours cluster in work settings and relate to burnout and safety culture: a cross-sectional survey analysis. *BMJ Qual Saf*. 2019;28(2):142-150. doi:10.1136/bmjqs-2018-007933

7. Adair KC, Rodriguez-Homs LG, Masoud S, Mosca PJ, Sexton JB. Gratitude at Work: Prospective Cohort Study of a Web-Based, Single-Exposure Well-Being Intervention for Health Care Workers. *J Med Internet Res*. 2020;22(5):e15562. doi:10.2196/15562

8. Rehder K, Adair KC, Sexton JB. The Science of Health Care Worker Burnout: Assessing and Improving Health Care Worker Well-Being. *Arch Pathol Lab Med*. 2021;145(9):1095-1109. doi:10.5858/arpa.2020-0557-RA

9. Profit J, Adair KC, Cui X, et al. Randomized controlled trial of the "WISER" intervention to reduce healthcare worker burnout. *J Perinatol*. 2021;41(9):2225-2234. doi:10.1038/s41372-021-01100-y

10. Sexton JB, Adair KC. Forty-five good things: a prospective pilot study of the Three Good Things well-being intervention in the USA for healthcare worker emotional exhaustion, depression, work-life balance and happiness. *BMJ Open*. 2019;9(3):e022695. doi:10.1136/bmjopen-2018-022695

© 2022 Sexton JB et al. *JAMA Network Open.*

11. Nunnally JC, Bernstein IH. *Psychometric Theory*. McGraw-Hill; 1994.

12. Nunnally JC. *Psychometric Theory*. McGraw-Hill; 1978.

13. James LR. Aggregation bias in estimates of perceptual agreement. *J Appl Psychol*. 1982;67(2):219-229. doi:10.1037/0021-9010.67.2.219

14. Smits M, Wagner C, Spreeuwenberg P, van der Wal G, Groenewegen PP. Measuring patient safety culture: an assessment of the clustering of responses at unit level and hospital level. *Qual Saf Health Care*. 2009;18(4):292-296. doi:10.1136/qshc.2007.025965

15. Bland JM. Sample size in guidelines trials. *Fam Pract*. 2000;17 Suppl 1:S17-20. doi:10.1093/fampra/17.suppl_1.s17

16. Park S, Lake ET. Multilevel modeling of a clustered continuous outcome: nurses' work hours and burnout. *Nurs Res*. 2005;54(6):406-413. doi:10.1097/00006199-200511000-00007

17. Bliese PD. Within-group agreement, non-independence, and reliability: Implications for data aggregation and analysis. In: *Multilevel Theory, Research, and Methods in Organizations: Foundations, Extensions, and New Directions*. Jossey-Bass; 2000:349-381.

© 2022 Sexton JB et al. *JAMA Network Open.*
